# Rupture of abdominal aortic aneurysm after intravitreal bevacizumab injection: a case report

**DOI:** 10.1186/1752-1947-8-48

**Published:** 2014-02-12

**Authors:** Sung UK Baek, Soon IL Kwon

**Affiliations:** 1 Department of Ophthalmology Hallym University Sacred Heart Hospital, #896 Pyeongchon-dong, Anyang-city, Gyeonggi-do 431-070, Republic of Korea

**Keywords:** Abdominal aortic aneurysm, Bevacizumab

## Abstract

**Introduction:**

We describe the case of a man who died of an abdominal aortic aneurysm rupture after an intravitreal injection of bevacizumab for neovascular age-related macular degeneration.

**Case presentation:**

A 74-year-old Korean man presented with visual disturbance in his right eye. He had previously been diagnosed with diabetes and hypertension, which were controlled with oral medications. We diagnosed him with neovascular age-related macular degeneration and he was treated by monthly intravitreal injection of bevacizumab for three months. Four days after his third intravitreal bevacizumab injection, he died of an abdominal aortic aneurysm rupture and uncontrolled bleeding.

**Conclusion:**

Abdominal aortic aneurysm rupture is highly lethal and there is a possible correlation with intravitreal injection of bevacizumab. Thus, we need to consider the risks of intravitreal bevacizumab injections for patients with abdominal aortic aneurysms.

## Introduction

Bevacizumab is a humanized recombinant monoclonal immunoglobulin G antibody that binds and inhibits all vascular endothelial growth factor (VEGF) isoforms. It has been used for the treatment of neovascular age-related macular degeneration (AMD), but was approved by the US Food and Drug Administration as an adjuvant agent in the treatment of metastatic colorectal carcinoma, not for neovascular AMD.

In chemotherapy regimens, bevacizumab is associated with an increased risk of thromboembolic events [1]; the systemic safety of an intravitreal injection of bevacizumab is unknown. There are only a few reports on adverse systemic effects, such as myocardial infarction, cerebrovascular accident and hypertension [2,3]. We report a case of abdominal aortic aneurysm rupture after an intravitreal bevacizumab injection for neovascular AMD.

## Case presentation

A 74-year-old Korean man presented with visual disturbance in his right eye. He had previously been diagnosed with diabetes and hypertension, which were controlled by oral medications.

**Figure 1:**
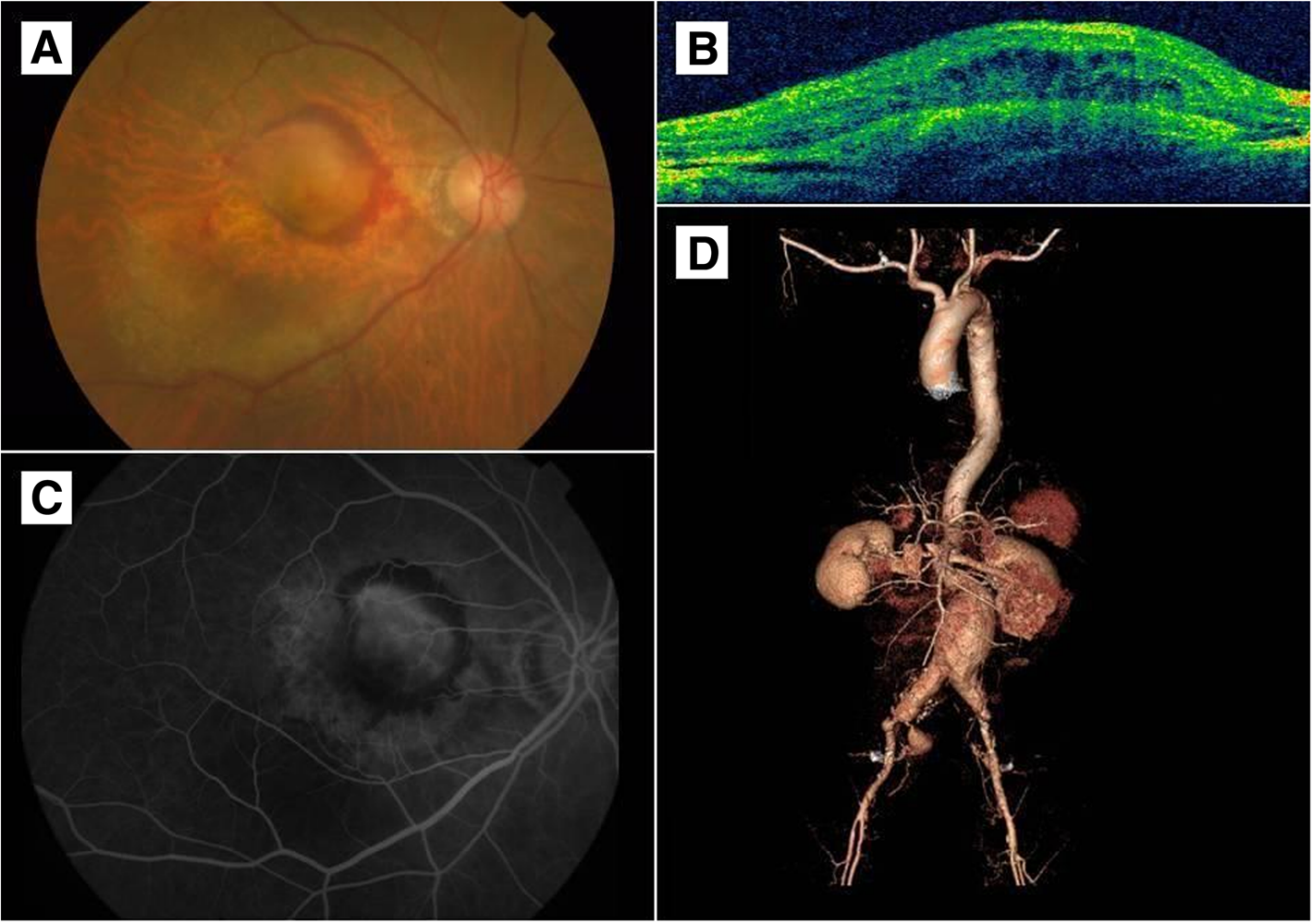
**Fundus photo, optical coherence tomography, fluorescein angiography and computed topography of patient. (A)** A fundus examination revealed macular elevations with subretinal neovascular membranes. **(B)** Optical coherence tomography showed submacular elevations with intraretinal edema. **(C)** Fluorescein angiography showed macular fluorescein leakage. **(D)** A contrast-enhanced computed tomography scan revealed an abdominal aortic aneurysm, 5.8cm in its largest diameter, with ruptured state and bilateral iliac artery aneurysms.

On his initial examination, his best corrected visual acuity was 20/40 in his right eye and 20/25 in his left eye. A fundus examination revealed macular elevations with a subretinal neovascular membrane in his right eye (Figure 1A). Optical coherence tomography showed submacular elevations with intraretinal edema (Figure 1B) and a fluorescein angiography showed macular fluorescein leakage in his right eye (Figure 1C). He was diagnosed with AMD.

We administered an intravitreal bevacizumab (1.25mg) injection for treatment of the AMD in his right eye. We used an aseptic technique, including irrigation of the conjunctival sac with diluted povidone-iodine (Betadine) solution. Our patient received oral and topical antibiotics for three days after the injection. The intravitreal injection of bevacizmab was to be repeated every month.

Four days after his third intravitreal bevacizumab injection, he visited our emergency room with decreased consciousness. On initial physical examination, his blood pressure was 83/54mmHg with a heart rate of 63 beats per minute. A contrast-enhanced computed tomography scan revealed an abdominal aortic aneurysm of 5.8cm in its largest diameter, with ruptured state and bilateral iliac artery aneurysms (Figure 1D). Although an emergency abdominal aortic aneurysm repair was performed, our patient died of uncontrolled bleeding through the ruptured wall.

## Discussion

AMD is the leading cause of irreversible blindness in people over the age of 50 in the developed world [4]. VEGF is considered a key player in the development of abnormal vessels for many pathological conditions, including AMD, and the antibodies that could inhibit the development of active VEGF forms [5,6].

Currently, the most commonly used VEGF antagonists are ranibizumab (Lucentis; Genentech, San Francisco, CA, USA) and bevacizumab (Avastin; Genentech, San Francisco, CA, USA). Ranibizumab has been approved for the treatment of patients with neovascular AMD by the US Food and Drug Administration. In contrast to ranibizumab, bevacizumab was not developed for the treatment of AMD and consequently has no approval for such use. But, because of its similar mode of action, availability and comparatively low costs, bevacizumab is widely used in the treatment of AMD [7,8].

In the several large randomized controlled trials of ranibizumab, the adverse systemic safety events that occurred during the study period were prospectively recorded, irrespective of their suspected relationships to the study treatments [9,10]. Systemic events caused by ranibizumab were rare. With bevacizumab, there are only a few reports of adverse systemic safety events, such as myocardial infarctions, cerebrovascular accidents and hypertension [2,3].

Bakri *et al.* reported the pharmacokinetics of 0.5mg of intravitreal ranibizumab (Lucentis) and 1.25mg of intravitreal bevacizumab (Avastin) using the same rabbit model [11]. After the intravitreal injection, no ranibizumab was detected in the serum but low concentrations of bevacizumab were detected. A maximum concentration of 3.3μg/mL was achieved eight days after the intravitreal injection, and the concentration had fallen below 1μg/mL 29 days after the intravitreal injection.

The intravitreal half-life of bevacizumab is estimated to be 5.6 days [12]. This is longer than that of ranibizumab, which is 3.2 days. So, repeated consecutive intravitreal injections of bevacizumab are more likely to result in accumulation than repeated ranibizumab injections.

There are various known predictors for an increased risk of abdominal aortic aneurysm rupture, such as the aneurysm size, aneurysm growth rate, smoking, chronic obstructive pulmonary disease and hypertension [13]. However, an association between chemotherapy and abdominal aortic aneurysm expansion has not yet been established. Palm *et al.*[14] reported a case of ruptured abdominal aortic aneurysm in a patient receiving chemotherapy with gemcitabine for pancreatic cancer. They suggested that the chemotherapy might have had disadvantageous effects on the abdominal aortic aneurysm. The potential mechanisms may have involved the inhibition of smooth muscle cell proliferation and collagen and elastin synthesis. But gemcitabine has different mechanisms from bevacizumab. Thus, this case is less relevant to ours.

In our patient, it seems reasonable to consider a possible correlation between intravitreal injection of bevacizumab and rupture of the abdominal aortic aneurysm. Injecting bevacizumab into the systemic circulation may have negative effects on hypertension and abdominal aortic aneurysm. Hypertension and instability of atherosclerotic plaque are well-known risk factors of abdominal aortic aneurysm rupture [15]. VEGF stimulates the synthesis of endothelial nitric oxide synthase and prostacyclin in endothelial cells, and its inhibition is associated with vasoconstriction that increases hypertension [16]. Also, bevacizumab inhibition of VEGF increases inflammation and atherosclerotic instability, which leads to plaque ruptures [17]. From our experience with our patient, we deduce that the two mechanisms could explain the occurrence of abdominal aortic aneurysm rupture.

## Conclusions

An intravitreal injection of bevacizumab in a patient with an abdominal aortic aneurysm is very rare. This makes any statistical analysis difficult, and it is unclear whether an abdominal aortic aneurysm rupture is an adverse effect of the intravitreal bevacizumab injection or an unrelated natural course of the aneurysm. But an abdominal aortic aneurysm rupture is highly lethal and there is a possible correlation with intravitreal injection of bevacizumab. Therefore, we need to consider the risks of intravitreal bevacizumab injection for patients with abdominal aortic aneurysms. To the best of our knowledge, this is the only case reported that describes large artery rupture that could be a result of an intravitreal bevacizumab injection.

## Consent

Written informed consent was obtained from the patient’s wife for publication of this case report. A copy of the written consent is available for review by the Editor-in-Chief of this journal.

## Abbreviations

AMD: age-related macular degeneration; VEGF: vascular endothelial growth factor.

## Competing interests

The authors declare that they have no competing interests

## Authors’ contributions

SUB and SIK managed our patient and wrote the preliminary draft of the manuscript. SUB was the major contributor in writing the manuscripts. Both authors read and approved the final manuscript.
